# Differences in Driving Anger among Professional Drivers: A Cross-Cultural Study

**DOI:** 10.3390/ijerph19074168

**Published:** 2022-03-31

**Authors:** Milanko Damjanović, Spasoje Mićić, Boško Matović, Dragan Jovanović, Aleksandar Bulajić

**Affiliations:** 1 Faculty of Mechanical Engineering, University of Montenegro, 81000 Podgorica, Montenegro; milanko@ucg.ac.me (M.D.); boskom@ucg.ac.me (B.M.); 2 Faculty of Transportation, Pan-European University “Apeiron”, 78000 Banja Luka, Bosnia and Herzegovina; spasoje.n.micic@apeiron-edu.eu; 3 Faculty of Technical Sciences, University of Novi Sad, 21000 Novi Sad, Serbia; draganj@uns.ac.rs; 4 Road Traffic Department, The Higher Education Technical School of Professional Studies, 21000 Novi Sad, Serbia

**Keywords:** driving anger, professional drivers, measurement invariance, aggressive driving, cross-cultural, public health, road transport

## Abstract

Public transport systems have a vital role in achieving sustainable mobility goals, diminishing reliance on private individual transport and improving overall public health. Despite that, transport operators are often in situations that require them to cope with complex working conditions that lead to negative emotions such as anger. The current study represents a segment of the permanent global research agenda that seeks to devise and test a psychometric scale for measuring driving anger in professional drivers. The present research is one of the first attempts to examine the factorial validity and the cross-cultural measurement equivalence of the broadly utilized Driving Anger Scale (DAS) in three culturally different countries within the Western Balkans region. The respondents (N = 1054) were taxi, bus and truck drivers between 19 and 75 years of age. The results pertaining to confirmatory factor analysis showed that there were adequate fit statistics for the specified six-dimensional measurement model of the DAS. The measurement invariance testing showed that the meaning and psychometric performance of driving anger and its facets are equivalent across countries and types of professional drivers. Furthermore, the results showed that driving anger facets had positive correlations with dysfunctional ways of expressing anger and negative correlations with the form of the prosocial anger expression. In addition, the results revealed that taxi drivers displayed considerably higher levels of anger while driving and aggressive driving than truck and bus drivers. Overall, this study replicates and extends the accumulated knowledge of previous investigations, suggesting that the original DAS remains a reliable and stable instrument for measuring driving anger in day-to-day driving conditions.

## 1. Introduction

Emotions that individuals experience in a specific situation may significantly affect their thoughts and behavioural reactions. One of the most common emotions that drivers experience in daily driving is anger. Anger is described as an approach-motivated affective state which is triggered in response to given negative valenced situational events [1]. Although it can be an adaptive emotion, high trait anger is in many instances accompanied by significant adverse outcomes [2,3,4]. For example, dysfunctional anger can negatively the cognitive ability and attentional processes [5,6], reduce behavioural inhibition [7], induce sarcasm [8] and vulnerable narcissism [9] or inflate the feeling of competence and cognitive ability [10]. Moreover, there is also evidence that uncontrolled anger may result in maladaptive behavioural consequences on the roads, such as aggressive and risky driving tendencies [11,12,13,14].

A great deal of research works have been devoted to empirically validating the concept of driving anger (DA) and determining its relevant dimensions, together with the implications with respect to road safety outcome performance. In a sample of American drivers, Deffenbacher et al. designed an instrument, the DAS, to assess the situations that evoke drivers’ feelings of anger when involved in the activity of driving [15]. The DAS is a 33-item scale that includes six correlated factors (i.e., “hostile gestures”, “traffic obstructions”, “discourtesy”, “police presence”, “slow driving” and “illegal driving”). Since the initial publication, the dimensionality of the DAS has attracted significant attention, and it has been examined in empirical research among different cultures. For example, it has been validated with datasets from the United Kingdom [16] Germany [17,18], France [19,20,21], Spain [22], Romania [23], Ukraine [24], Serbia [11], Turkey [25], Argentina [26], New Zealand [27,28], Malaysia [29] and China [30,31,32]. The vast majority of the above-mentioned research has shown that the original six-factor solution had adequate model fit indices, particularly if similar item pairs were covaried or if some of the items were eliminated as a result of factor loadings that were low or problems regarding cross-loadings [17,19,22,24,28,29,30]. However, there are few research works that have failed to reproduce the original six-factor structure [11,16,20,21,26,27]. This may imply that the structure relying on six factors is unstable or the items may operate variously throughout several research works, target groups, languages and cross-cultural settings, and it could be beneficial to examine further model robustness.

Therefore, it remains an open psychometric issue whether the meaning of DA is understood identically by drivers within diverse sociocultural contexts and target groups. In order to respond to this specific issue, it is essential to conduct comparative analyses to ascertain whether the meaning and psychometric performance of DA and its facets are invariant across these heterogeneous settings. Although the DAS has been employed among more than 20 cultures [33], based on our experience, an insufficient body of research examines its cross-cultural validation. In addition, the conceptual framework of the DAS initiated by Deffenbacher et al. [15] has not been validated among professional drivers in the countries of the Western Balkans (i.e., Serbia, Bosnia and Herzegovina and Montenegro). The further examination of the psychometric performance of the DAS in these cultures and specific groups of drivers will enhance its validity.

At first glance, it is difficult to notice the essential disparities that exist between the three countries of the Western Balkans. Despite some regional similarities such as language, history and geography [34], there also are substantial differences which could potentially affect the degree of measurement invariance. After the collapse of former Yugoslavia, ethnic identity became a prominent contextual factor which enhanced the intercultural communication gap between the three former member states [35]. Today, a fairly large number of Serbs live in Serbia (RS), but there are also a substantial number of Serbs in neighbouring Montenegro (MNE) and Bosnia and Herzegovina (B&H). In the same way, the greatest number of people from B&H are Bosniaks, but similarly, they constitute the minority in the neighbouring RS and MNE. In addition, the highest proportion of Montenegro’s population are Montenegrins, whereas they are recognized as a minority ethnic group in the neighbouring RS and B&H [35]. Moreover, ethnicity in these groups of countries is accompanied by religious differences: the Serbs are Orthodox believers, the Bosniaks are Muslims, while the Montenegrins are mainly Orthodox and Muslims [35]. As noted by Lau et al. [36], religious affiliations might be a potential source of the distortion of measurement invariance. There are also some national differences in terms of Hofstede’s [37] cultural dimensions that might have a key role when investigating DA. As identified by Hofstede, B&H is a more indulgent culture in comparison with the other two cultures. Likewise, RS is more focused on a short-term future perspective, contrary to MNE and B&H, which are more oriented towards a long-term future perspective [38]. There are also substantial discrepancies across the three Western Balkan countries in terms of indicators pertaining to road safety, as estimated by the rate of traffic deaths per 100,000 people. In 2016, RS had a mortality rate of 7.4 deaths per 100,000 people, making it approximately two times lower than in B&H (i.e., 15.7 deaths per 100,000 people) and roughly 1.5 times lower than in MNE (i.e., 10.7 deaths per 100,000 people) [39]. The previous studies [40,41,42] pointed out the existence of close relationships between the society’s cultures and road safety, which in turn might result in a lack of measurement invariance among countries with a different level of road safety performance. Therefore, accurate comparative conclusions need evidence of factorial invariance to prove that systematic variation exists in the outcome variable due to systematic factors rather than measurement biases. Nevertheless, insufficient data are available on whether examining the measurement equivalence between socially close nations may lead to a greater level of comparability.

Furthermore, the previous studies have failed to provide empirical evidence of the stability and replicability of the original DAS structure among professional drivers [17,31]. For instance, Brandenburg et al. [17] found that the original DAS was not appropriate to measure DA in professional German taxi drivers, since the model fit indices have been fairly poor. Similar results were reported in a study among bus drivers in China [31], which revealed that a four-factor solution containing 19 items is more adequate than the original six-factor model. These inconsistencies in the results may indicate that the six-factor solution validated in non-professional drivers was not an adequate representation of the DAS factor structure among professional drivers. Accordingly, this may imply that the original six-factor model did not seem to support configural invariance of the DAS among professional drivers. In addition, little knowledge exists on the measurement equivalence of the original DAS across taxi, truck and bus drivers around the world. Hence, empirical research is necessary to ensure the measurement equivalence of constructs among professional drivers as an essential precondition for the further comparison of the means of latent variables and correlations across different groups.

The majority of previous research has given an insight into the good empirical evidence in relation to the convergent validity of the DAS facets. For instance, the meta-analytic studies performed over the last two decades indicated that DA correlates moderately and positively with aggressive driving [43,44,45,46]. Furthermore, in a study by Dahlen et al. [47], anger pertaining to driving had a positive correlation to the three types of the dysfunctional expression of DA (i.e., “verbal aggression”, “using vehicles to express anger” and “physical aggression”). Conversely, DA had a negative correlation to the adaptive DA expression [21,44,45,48]. Regarding the DAS subscales, “hostile gestures”, “traffic obstructions”, “discourtesy”, “police presence” and “slow driving” displayed a positive correlation to the expressions of DA that were aggressive in nature. On the other hand, a negative correlation could be observed in relation to the non-aggressive expression. However, contrary to expectations, the insignificant and/or inverse correlations between illegal driving and aggressive driving were revealed across prior studies [21,48]. The previous research revealed a weak positive association between DA and the involvement in traffic violations and accidents [44,45,46,49]. In addition, all the DAS subscales were positively correlated with these adverse driving outcomes except illegal driving, which was correlated negatively [20,28,48].

A large body of prior research has been performed in order to establish the differences that exist between diverse types of professional drivers in terms of their unsafe driving behaviours which require change [50,51,52]. For instance, Huang et al. [50] revealed that taxi drivers had a higher degree of susceptibility to displaying aberrant driving as opposed to bus drivers. Similar conclusions were arrived at by Mehdizadeh et al. [51], who revealed that taxi drivers had a higher likelihood of engaging in driving behaviour of a risky nature (i.e., errors, aggressive as well as ordinary violations) than truck drivers. Moreover, Öz et al. [52] emphasized that minibus drivers had a tendency to commit more traffic violations than heavy vehicle drivers. Nevertheless, their results also revealed the absence of significant differences in aggressive behaviours between divergent categories of professional drivers. Previous empirical research focusing on DA experience and expression has mainly been performed within a particular type of professional drivers, such as truck drivers [30,31,53]. The fact that the comparison between various types of professional drivers regarding these components has attracted insufficient attention in prior research indicates the need for the present study to bridge the gap mentioned above.

### The Present Study

The primary purpose of the current research was to assess the underlying structure and the cross-cultural measurement invariance of the broadly utilized DAS instrument established by Deffenbacher et al. [15] among professional drivers in the Western Balkan region. More concretely, the goals were the following: (1) to examine the underlying factorial structure of the initial six-factor DAS model in the three Western Balkan countries; (2) to test the measurement equivalence pertaining to the six-factor model used for measuring the DAS within the three countries and groups of professional drivers; (3) to investigate whether the DAS subscales are associated in the expected direction with aggressive and non-aggressive driving behaviour, as well as with reported traffic violations and road accidents; (4) to compare the degree of DA across the three countries and categories of professional drivers.

The remainder of this paper is structured as follows. Section 2 refers to the methodology, outlines the utilized data and provides a description of the multivariate techniques used to analyse data. Section 3 then presents the results of the examination of the psychometric properties of the DAS and comparison analysis between various types of professional drivers and different cross-cultural environments. Section 4 discusses the main results in the context of the existing literature. Section 5 concludes the paper and discusses the theoretical and practical implications.

## 2. Materials and Methods

### 2.1. Participants

The study comprised a dataset of 1054 drivers who hold a professional driving license from RS (*n* = 313), MNE (*n* = 440) and B&H (*n* = 301). The sample consisted of 331 taxi drivers, 328 bus drivers and 395 truck drivers. The participants spanned between 19 and 75 years old (M = 43.2; SD = 10.9). The major proportion of participants were male (98.8%) and had completed secondary school (88.9%). The professional driving experience ranged between 1 year and 50 years (M = 19.3; SD = 11.0). The number of kilometres driven in the past 12 months ranged between 4000 and 180,000 (M = 67,456.4; SD = 38,353.8). More than half of the participants (55.4%) stated having experienced a minimum of one driving violation in the last three years. In addition, nearly one third of the participants (29.6%) reported engagement in one or more road accidents within the past three years. Table 1 gives an insight into the socio-demographic and driving data of professional drivers. Further details on the sample composition by countries are given in Supplementary Materials (see Tables S1–S3).

### 2.2. Measures

The questionnaire utilized in the present research was translated into target languages using the common forward–backward–forward translation method proposed by Brislin [54]. The process of translating the questionnaire was initially conducted by the authors. Next, a professional language editor for each language reviewed the linguistic validity of the translated questionnaire. Any content discrepancies identified in this process were further considered and reconciled. Furthermore, the questionnaire was again translated into the English language on the part of another three separate bilingual experts. Finally, the authors, along with the professional translators, examined and matched the translated version of the questionnaire with the original one, and any differences were addressed. The main body of the questionnaire consisted of three sections, including the background information of the participants, propensity to experience anger during the act of driving, as well as anger expression that was aggressive when driving.

The background information of the participants including socio-demographic variables (for instance, age, gender and education attainment), attributes pertaining to driving (for instance, yearly mileage as well as professional driving experience), driving violation history (i.e., involvement in at least one driving violation in the last three years) and road accident data (i.e., involvement in at least one road accident in the last three years) were collected. 

The DAS [15] is an instrument designed to assess drivers’ susceptibility to experiencing anger that can be observed within a traffic environment. The original DAS was made up of 33 self-report items that assess the six domains of anger-inducing situations. “Hostile gestures” (HG) were assessed using three indicators (e.g., “Someone shouts at you about your driving”.). “Illegal driving” (ID) was measured through four indicators (e.g., “Someone runs a red light or stop sign”.). “Police presence” (PP) was assessed by four indicators (e.g., “You pass a radar speed trap”.). “Slow driving” (SD) was measured by six indicators (e.g., “Someone is slow in parking and holds up traffic”.). “Discourtesy” (Dis) was estimated through nine indicators (e.g., “Someone speeds up when you try to pass them”.). “Traffic obstruction” (TO) was measured using seven indicators (e.g., “You are stuck in a traffic jam”.). Respondents answered all the questions using a five-point Likert scale from not at all (1) to very much (5). 

A shortened type of the Driving Anger Expression Inventory (DAX-short) [55] was applied with the aim of measuring drivers’ tendencies to display anger during the act of driving. The DAX-short is a 15-item instrument that comprises three forms of aggressive driving behaviour and one form of prosocial driving behaviour. The “verbal aggressive expression” (Ver) was assessed using three indicators (e.g., “I yell at other drivers”.). The “personal physical aggressive expression” (Phy) was measured by four indicators (e.g., “I try to scare other drivers”.). The “use of the vehicle to express anger” (Veh) was assessed by three indicators (e.g., “I drive right up to the other driver’s bumper”.). The “adaptive/constructive expression” (Adp) was measured by five indicators (e.g., “I tell myself to ignore it”.). The participants provided answers to the questions by means of applying a five-point Likert scale from never (1) to always (5). Previous research conducted in Serbia confirmed appropriate internal consistency reliability for each of the four dimensions of the DAX, ranging from 0.77 to 0.88 [56]. More details with respect to the psychometric performance of the DAX-short are provided in Supplementary Materials (see Tables S4–S6).

### 2.3. Procedures

The study was briefly introduced over the phone to numerous managing directors of transport companies in each country. When some of them accepted to cooperate, we phoned the transport managers and requested that they distribute the questionnaires to professional drivers employed in the transport sector. With their assistance, the questionnaires were administered in the paper-and-pencil version. The participants were guaranteed that the answers they provided were going to be treated anonymously as well as confidentially. They were also told that they were cooperating on a voluntary basis. Those who agreed to participate gave written informed consent and spent roughly 30 min in the workplace to fill out a questionnaire battery. In order to ensure their privacy, the participants inserted a completed questionnaire into a drop box, which was placed in each company. They were given the details regarding their ethical rights and the chance to withdraw their consent any time they wanted to. Overall, out of the 1500 questionnaires that were administered, 1067 were received back, leading to a 71.1% response rate. The design of the present research received the approval of the Ethics Committee of University of Montenegro in 2020.

### 2.4. Statistical Analyses

The data analyses were systematized in six steps. Firstly, the preliminary data assessment was carried out with the aim of identifying the missing data, extreme values and normality of data using IBM SPSS Statistics (V26). In addition, descriptive statistics (i.e., measures of dispersion, central tendency, symmetry and the shape of the distribution) were employed to summarize the sets of data. The univariate normality of data was assessed by skewness and kurtosis values. When these values are close to zero, the distribution is considered to be normal [57]. Secondly, the factorial structure of the original DAS for each particular country was estimated by means of the Confirmatory Factor Analysis (CFA) in R “lavaan” package [58]. The multivariate normality was checked using the Mardia test in the “semTools” package in R [59]. When the Mardia coefficient is above the critical threshold of p(p+2), where p refers to the number of existing indicators, then non-normality can be assumed in the mixed distribution of the variables [60]. Because of the ordinal nature of the indicators and moderate deviations from multivariate normality, the Diagonally Weighted Least Squares (DWLS) estimator was applied [60,61]. Fitting the model to the data was evaluated by means of several goodness-of-fit (GoF) indices: the Chi-square statistic, the Root Mean Square Error of Approximation (RMSEA) with a confidence interval that is 90%, the Tucker–Lewis Index (TLI), the Comparative Fit Index (CFI) and the Standardized Root Mean Square (SRMR). According to the guidelines that are used to interpret GoF indices available in the existing literature [62,63,64,65], an RMSEA scores less than 0.05 suggests a very good fit, scores within the limits of 0.05–0.08 imply an acceptable fit, and scores within 0.08–0.10 show the presence of a fit that is mediocre. In addition, scores exceeding 0.10 imply a poor fit. SRMR scores lower than 0.05 display a good fit, and scores within 0.05 and 0.08 show an adequate fit. CFI and TLI scores exceeding 0.90 show that there is an acceptable fit when it comes to the dana, and scores of at least 0.95 display a good fit. According to Tabachnick and Fidell [57], standardized loading estimates higher than or equal to 0.32 should be considered reasonable. Thirdly, a multi-group confirmatory factor analysis (MGCFA) was carried out to assess the invariance related to the proposed six-factor model across the three countries and professional driver types (i.e., taxi, bus and truck drivers) by utilizing the “semTools” package in R [59]. Testing measurement invariance contains a comparison of several models that enforce consecutive equality constraints on the model parameters (i.e., configural, metric, scalar and residual). Configural invariance implies the intergroup equivalence of the model form (i.e., baseline unconstrained model). Metric invariance refers to the intergroup equality of factor loadings, while scalar invariance additionally assumes the intergroup equivalence of item intercepts, and a residual invariance which, in addition, assumes the intergroup equivalence of item residuals (i.e., the constrained nested model). Factorial invariance was assessed by using the chi-square difference (i.e., ΔDWLS χ^2^) between the two nested models, whose adequacy had previously been criticized because of the susceptibility of the chi-square statistics to the number of sample cases [66]. Therefore, the model’s invariance across groups was estimated by comparing the difference between fit statistics (i.e., ΔCFI and ΔRMSEA) for the two nested models. According to the most commonly used criteria, changes in the CFA < 0.02 and changes in the RMSEA ≤ 0.015 indicate that the assumptions associated with invariance across groups cannot be rejected [66,67,68,69]. Fourthly, internal consistency reliability was computed by means of applying Chronbach’s alpha [70] and Raykov’s omega coefficient [71] in the “semTools” package in R [59]. The value of these indicators is recommended to be higher than the acceptability threshold of 0.70. Fifthly, Spearman’s correlation coefficients were applied when testing the convergent validity of the subscales in R package “psych” [72]. Convergent validity could be identified in those cases when significant and meaningful correlations existed at the level of DAS domains, DAX-short domains, driving violation history and road accident data. In line with the general guidelines, the magnitude of correlations was classified as weak when 0.1 ≤ ρ < 0.3, moderate when 0.3 ≤ ρ < 0.5 and strong when ρ > 0.5 [73]. Lastly, because of the uneven sample group sizes and violations of the normality assumption, non-parametric methods (i.e., Kruskal–Wallis and Wilcoxon Rank Sum tests) were employed to compare the scores on the DAS subscales across countries and professional driver types. In order to determine the strength of the association between variables, a standardized measure of this magnitude, known as the effect size, was computed. Effect sizes values can range from 0 (no effect) to 1 (perfect effect). According to Tabachnick and Fidell [57], values less than 0.10 suggest a small effect, values within 0.11–0.30 imply a medium effect, and values greater than 0.50 show a large effect. To prevent type 1 error inflation, due to the multiple comparisons, Bonferroni corrections were applied. Statistical analyses were performed using the “rstatix” package in R [74].

## 3. Results

### 3.1. Preparatory Analyses and Summary Statistics

Before conducting the main analyses, a preliminary data assessment was conducted. Of the 1054 respondents, 1.1% had one missing value. The multiple imputation technique with five imputations was employed to deal with missing data. This imputation was performed using IBM SPSS Statistics (V26). Furthermore, univariate extreme values (z > ±3.33) were removed in all datasets independently (four from RS, six from MNE and three from B&H). The findings pertaining to the descriptive statistics of the DAS and the DAX-short measures by countries are displayed in Table 2. These results indicated that professional drivers in all countries were prone to displaying more pronounced levels of anger when encountering discourteous drivers, as well as showing their anger in a verbally aggressive way and using their vehicle to react to anger-provoking situations. Inspections of skewness and kurtosis values across three countries indicated that data were generally non-normally distributed. In addition, Mardia’s tests demonstrated a significant violation of the multivariate normality of the data obtained within the countries (see Table 2). More information regarding the descriptive statistics of the DAS and the DAX-short scales is available in Supplementary Materials (see Tables S7–S9).

### 3.2. Confirmatory Factor Analyses

The results of the CFA are presented in Table 3 and Table 4. The indices of fit for the hypothesized original measurement model across countries were within acceptable to very good limits, except for the SRMR in B&H, which was slightly greater than the proposed minimum threshold of acceptance (see Table 3). All indicator variables had a significant loading on their own particular general factor (*p* < 0.001). As indicated in Table 4, the standardized estimates for the Dis subscale ranged from 0.526 to 0.751 across countries. In terms of the HG subscale, all indicators had factor loadings above 0.670 in each country. The factor loadings referring to the ID subscale spanned from 0.530 to 0.817 in the three countries. With respect to the PP subscale, the standardized estimates for the majority of the indicators were above 0.510 in all countries. The factor loadings for the SD subscale were in the range of between 0.467 and 0.757 across samples. The examination of factor loadings for the TO subscale showed that indicator 31 (i.e., “encountering road construction zones”) had the smallest value of 0.370 in the Montenegrin sample and 0.422 in the Bosnian sample, but this was still higher than the recommended 0.32 threshold.

### 3.3. Multi-Group Analysis of Invariance

Table 5 presents the results of the MG-CFAs which were conducted to estimate the measurement invariance associated with the six-factor model of the DAS in the observed countries, along with the types of professional drivers. An inspection of the model fit criteria used to assess configural invariance revealed an identical factor structure that was supported across all countries (CFI = 0.975). When invariance constraints were imposed on factor loadings, the value of the ΔDWLS χ^2^ = 753.96 (Δdf = 54), *p* < 0.001 was significant. However, the metric model indicated an acceptable shift in the alternative GoF indices (ΔCFI = 0.014; ΔRMSEA = 0.011), indicating that factor loadings are equal across countries. Constraining the item intercepts aside from factor loadings led to a significant change in the value of the ΔDWLS χ^2^ = 228.72 (Δdf = 54), *p* < 0.001. Similar to before, an acceptable shift in the alternative GoF indices (ΔCFI = 0.004; ΔRMSEA = 0.001) was identified, implying that scalar invariance is confirmed for the original DAS. Imposing equality constraints on the item residuals led to a significant change in the score of the ΔDWLS χ^2^ = 196.45 (Δdf = 66), *p* < 0.001. Once again, the shift in alternative GoF indices (ΔCFI = 0.003; ΔRMSEA = 0.001) was considerably below the cut-off criteria, indicating that the item residuals are equal across all countries. 

Further, the results displayed in Table 5 show support for the configural invariance between taxi, bus and truck drivers (CFI = 0.964). When the model with constrained factor loadings is compared to the unconstrained baseline model, the shift in the alternative GoF indices was acceptable (ΔCFI = 0.010; ΔRMSEA = 0.006), although the difference value of the ΔDWLS χ^2^ = 491.47 (Δdf = 54), *p* < 0.001 was significant, thus suggesting that factor loadings are equal across the types of professional drivers. In addition, a significant change in the value of the ΔDWLS χ^2^ = 77.93 (Δdf = 54), *p* < 0.05 was identified after introducing equality restrictions on the intercepts. However, the scalar model showed an acceptable shift in the alternative GoF indices (ΔCFI = 0.001; ΔRMSEA = 0.001), suggesting that the item intercepts are invariant across the three types of professional drivers. Finally, constraining the item residuals yielded an acceptable shift in alternative GoF indices (ΔCFI = 0.006; ΔRMSEA = 0.003). Although the difference value of the ΔDWLS χ^2^ = 330.20 (Δdf = 66), *p* < 0.001 was significant, the findings provide evidence for the residual invariance model.

On the whole, these findings suggested that the six-factor model of the DAS showed strong configural and measurement invariance across three countries in the Western Balkan region and types of professional drivers, with equivalence of the factor structure, factor loadings, item intercepts and item residuals. 

### 3.4. Internal Consistency Evaluation

The results of the internal reliability investigation are displayed in Table 2. The internal consistency reliability indices for the DAS subscales were all acceptable across countries. In addition, the internal reliability indicators for the DAX-short subscales were higher than the minimum acceptable threshold in all countries with the exception of the Phy subscale in MNE and B&H. 

### 3.5. Convergent Validity

The correlation coefficients (Spearman) were calculated between the six DA experience and four DA expression subscales, using the pooled sample (see Table 6). As expected, the results indicated that all DA measures were positively correlated, albeit weakly to moderately, with three types of DA expressions that were aggressive in nature (i.e., Ver, Phy and Veh). At the same time, the three DA domains (i.e., HG, PP and SD) correlated weakly and negatively with the subscale of the prosocial DA expression (i.e., adaptive/constructive). Conversely, the ID subscale tended to correlate weakly and positively with the Adp subscale. In addition, Dis, TO, PP and SD have a tendency to correlate positively and weakly with self-reported traffic violations, and the HG subscale correlated weakly and positively with self-reported road accidents.

### 3.6. Group Comparisons

Table 2 presents the results of the non-parametric Kruskal–Wallis and Wilcoxon multiple comparison tests, which were conducted to compare professional drivers’ scores across the three countries on two scales related to experience as well as expressing anger in the act of driving. The results indicated that three of the six DAS domains were significantly different across the three countries. Accordingly, the professional drivers’ nationality significantly affected their DA level due to discourteous drivers (*χ*^2^ = 6.24, df = 2, *p* < 0.05). The professional drivers from MNE stated that they had experienced a lower level of Dis in relation to the drivers who came from B&H (*Z_kw_* = −2.00, *p* < 0.05, *r* = 0.09). Furthermore, professional drivers significantly differed as a function of nationality with regard to illegal driving (χ^2^ = 12.53, df = 2, *p* < 0.01). Montenegrin professional drivers had a significantly higher score on the ID subscale than Serbian drivers (*Z_kw_* = −3.29, *p* < 0.05, *r* = 0.13). The differences in the scores on the TO domain were significant (χ^2^ = 31.66, df = 2, *p* < 0.001). Serbian professional drivers had a higher score on the TO subscale than Montenegrin drivers (*Z_kw_* = 4.70, *p* < 0.001, *r* = 0.18) and Bosnian (*Z_kw_* = 4.71, *p* < 0.001, *r* = 0.20) drivers. There were no differences that could identified across countries in terms of HG, PP and SD domains. 

Concerning the verbally aggressive DA expression, there was a significant main effect of nationality (χ^2^ = 24.80, df = 2, *p* < 0.001). Professional drivers from Serbia scored significantly higher on the Ver subscale than those from MNE (*Z_kw_* = 4.73, *p* < 0.001, *r* = 0.18) and B&H (*Z_kw_* = 2.65, *p* < 0.01, *r* = 0.12). Moreover, professional drivers significantly differed as a function of the country of residence with respect to the Phy expression (χ^2^ = 10.24, df = 2, *p* < 0.01). Serbian professional drivers scored significantly higher on the Phy subscale than Bosnian drivers (*Z_kw_* = 2.88, *p* < 0.01, *r* = 0.13). In addition, the differences in the scores on the Veh domain were significant (χ^2^ = 12.30, df = 2, *p* < 0.01). Serbian professional drivers had a higher score on this subscale than Montenegrin drivers (*Z_kw_* = 2.14, *p* < 0.05, *r* = 0.09), and Montenegrin motorists had a higher score on this subscale than Bosnian (*Z_kw_* = 2.97, *p* < 0.01, *r* = 0.12) drivers. Finally, the professional drivers’ nationality significantly affected their adaptive constructive ways to regulate DA (χ^2^ = 11.51, df = 2, *p* < 0.01). Professional drivers from MNE scored significantly higher on the Adp subscale than those from Serbia (*Z_kw_* = −2.07, *p* < 0.05, *r* = 0.09) as well as B&H (*Z_kw_* = 2.81, *p* < 0.01, *r* = 0.12).

Table 7 reports the findings of descriptive statistics and the non-parametric Kruskal–Wallis tests for the DAS and DAX-short measures among the types of professional drivers. The differences in the scores on the Dis domain were significant (χ^2^ = 65.88, df = 2, *p* < 0.001). Taxi drivers had a significantly higher score on the Dis subscale than bus drivers (*Z_kw_* = 7.73, *p* < 0.001, *r* = 0.31) as well as truckers (*Z_kw_* = 3.09, *p* < 0.01, *r* = 0.13), and bus drivers had a significantly lower score on this subscale than truck drivers (*Z_kw_* = −5.05, *p* < 0.001, *r* = 0.20). Regarding the HG domain, a significant main effect of the professional driver types (χ^2^ = 24.85, df = 2, *p* < 0.001) was identified. Bus drivers had a score that was considerably lower on the HG subscale than taxi drivers (*Z_kw_* = 4.51, *p* < 0.001, *r* = 0.18) and truckers (*Z_kw_* = −3.82, *p* < 0.001, *r* = 0.15). Furthermore, the professional drivers’ types significantly affected their DA level because of the illegal behaviour of the other drivers (χ^2^ = 27.41, df = 2, *p* < 0.001). Bus drivers had considerably lower results observed on the ID subscale than taxi drivers (*Z_kw_* = 2.97, *p* < 0.01, *r* = 0.13) and truckers (*Z_kw_* = −5.05, *p* < 0.001, *r* = 0.20). In addition, professional drivers significantly differed as a function of their driving licence category in respect to the PP domain (χ^2^ = 67.59, df = 2, *p* < 0.001). When it comes to bus drivers, their level of DA was lower due to the presence of the police than taxi drivers (*Z_kw_* = 8.00, *p* < 0.001, *r* = 0.32) and truckers (*Z_kw_* = −5.48, *p* < 0.001, *r* = 0.21), and taxi drivers displayed higher levels of anger when driving in these situations compared to truck drivers (*Z_kw_* = 2.21, *p* < 0.05, *r* = 0.10). In addition, the results revealed a significant main effect of the professional driver types on DA experience due to slow drivers (χ^2^ = 97.45, df = 2, *p* < 0.001). Taxi drivers had a significantly higher score on the SD subscale than bus drivers (*Z_kw_* = 9.44, *p* < 0.001, *r* = 0.32) as well as truckers (*Z_kw_* = 5.47, *p* < 0.001, *r* = 0.21), and bus operators had a lower score on this subscale compared to truck drivers (*Z_kw_* = −4.98, *p* < 0.001, *r* = 0.19). The differences in the scores on the TO domain were also significant (χ^2^ = 86.40, df = 2, *p* < 0.001). Taxi drivers scored significantly higher on the TO subscale than bus operators (*Z_kw_* = 8.83, *p* < 0.001, *r* = 0.35) and truckers (*Z_kw_* = 4.14, *p* < 0.001, *r* = 0.16), and bus operators scored significantly lower on this subscale than truckers (*Z_kw_* = −5.63, *p* < 0.001, *r* = 0.22). 

Further, professional drivers significantly differed as a function of their driving licence category with regard to verbal aggression (χ^2^ = 34.60, df = 2, *p* < 0.001). Taxi drivers had a significantly higher score on the Ver subscale than bus drivers (*Z_kw_* = 5.58, *p* < 0.001, *r* = 0.23) and truckers (*Z_kw_* = 2.26, *p* < 0.05, *r* = 0.10), and bus operators had a lower score on this subscale compared to truck drivers (*Z_kw_* = −3.35, *p* < 0.001, *r* = 0.14). With regards to the Phy domain, a significant main effect of the professional driver types (χ^2^ = 23.00, df = 2, *p* < 0.001) was detected. With respect to bus drivers, their physical aggressive DA expression proved to be at a lower level as opposed to taxi drivers (*Z_kw_* = 4.34, *p* < 0.001, *r* = 0.18) and truckers (*Z_kw_* = −3.44, *p* < 0.001, *r* = 0.14). The differences in the scores on the Veh subscale also proved to be significant (χ^2^ = 27.47, df = 2, *p* < 0.001). Taxi drivers scored significantly higher on the Veh subscale than bus drivers (*Z_kw_* = 5.05, *p* < 0.001, *r* = 0.21) and truckers (*Z_kw_* = 2.26, *p* < 0.05, *r* = 0.10), and bus operators scored significantly lower on this subscale than truck drivers (*Z_kw_* = −2.41, *p* < 0.05, *r* = 0.10). Lastly, the differences in the scores on the Adp domain were significant (χ^2^ = 6.75, df = 2, *p* < 0.05). Taxi drivers had a significantly higher score on this subscale than bus drivers (*Z_kw_* = 2.03, *p* < 0.05, *r* = 0.10). 

## 4. Discussion

The current study represents a segment of the permanent global research agenda that seeks to devise as well as test a psychometric scale used for measuring DA. Although DA has been an important research topic for more than two decades, little information is available on the psychometric validation of the DAS across different cultural settings and target groups. To address this gap, this research sought to establish a cross-culturally valid and reliable instrument to assess the extent of DA present in professional drivers. It should be noted that the findings regarding confirmatory factor analysis showed that there were adequate fit statistics for the specified six-dimensional measurement model of the DAS in all samples. The findings of this study offered evidence that suggested the existence of invariance in factors pertaining to structure, loadings, item intercepts as well as item residuals among professional drivers from culturally different environments and categories of driving licences. The present study confirmed the findings about the reliability and convergent validity of the DAS. A comparison analysis indicated that taxi drivers possessed a higher level of experience and expression of DA than truck and bus drivers. 

In this research, we utilized factor analysis methods to explore the dimensionality of the original conceptual framework of the DAS. The findings obtained in the current study proved that the six-dimensional factor model gave good fit indices across samples from three culturally different countries within the Western Balkans region: Montenegro, Bosnia and Herzegovina and Serbia. These results proved to be congruent with previous research and confirmed the validity of the six-factor model of the DAS [17,19,22,24,28,29,30]. The research findings showed that the factor loadings of indicator 31 (“encounter road construction and detours”) are lower than the others. However, the factor loadings on this indicator were higher than the recommended cut-off value. Recent studies have shown that this indicator had lower factor loadings compared to other indicators [18,19,30], which suggests that it might not be appropriate for capturing situations in which professional drivers experience anger due to TO. In addition, these situations are not common in traffic, and therefore they may not adequately reflect everyday traffic obstructions. 

Within the study at hand, an analysis of the measurement invariance was conducted to explore the six-factor measurement model of the DAS among professional drivers across the three countries. The research findings showed that the DAS is configurally invariant, indicating that the basic dimensional structure of the DAS was supported in all the countries and types of professional drivers. These findings add to the existing knowledge on the stability of the factor structure revealed within the original datasets from the US [15] and its further replicability identified by several succeeding factor analytic studies [17,19,22,24,28,29,30]. Furthermore, the present data provided empirical confirmations of the metric invariance of the DAS, indicating that the facets of DA have a similar meaning among professional drivers across the three nations. Although the Western Balkan countries are culturally heterogeneous, they have a similar language background. Previous research findings [75] have indicated that linguistic similarity may serve as an indicator for cultural closeness between countries and impact the degree of measurement equivalence. Therefore, the present findings should be regarded as initial evidence for the cross-country metric invariance of the DAS. The cross-language measurement equivalence of the DAS should be examined by further studies including more linguistically diverse countries. Moreover, the study results provided evidence for the presence of scalar invariance of the DAS, which suggests that the means of the DAS constructs can be validly compared between the three countries and types of professional drivers. Finally, these results also indicated that a strict form of invariance across countries and professional driver types was tenable, providing support for a reasonable comparison in latent factor means and the magnitude of correlation/regression coefficients across these groups. Overall, the results of the current research showed that full configural and measurement invariance were established, providing consistency in measurement across samples as an important prerequisite for a further comparison analysis. 

In the current study, convergent validity was evaluated by considering the correlations between DA experience measures, DA expression measures, self-reported traffic violations and road accidents. The findings pertaining to the study at hand showed that the convergent validity of the DAS was supported. The expected direction of relationships between almost all the measures was identified. The size of the correlation coefficients ranged from weak to moderate. This substantiates the previous findings, indicating that the six different types of DA had positive correlations with dysfunctional forms of DA expression and negative correlations with the form of the prosocial DA expression [21,44,45,47,48]. This means that drivers who experienced higher degrees of DA while driving expressed more aggression through verbal and physical forms and less in an adaptive way. The inspection of the correlation coefficients between the DAS subscales and traffic violations indicated the existence of weak and positive relationships. Concerning the association of the six different forms of DA with road accidents, a weak positive correlation was identified only for HG. The direction and strength of relationships are in line with the findings obtained in prior studies [44,45,46,49]. Altogether, the correlation results underpin the convergent validity of the DAS among professional drivers.

In the last stage of the analysis, non-parametric comparison tests were conducted to assess the differences in anger experience and expression across the various types of professional drivers and countries. This study found that Serbian professional drivers had a higher level of anger in situations that frustrate or impede them, such as traffic congestion or weak road infrastructure. Prior studies have found that poor road conditions could evoke higher levels of driving anger [24]. In this study, it appears that Serbian professional drivers had a higher tendency to engage in aggressive behaviours than Montenegrin drivers and Bosnian drivers. This could be attributed to the fact that Serbian culture is more focused on a short-term future orientation compared to the other two. This is consistent with previous studies that indicated that short-term orientation cultures are associated with relatively greater levels of aggression and anger expression [76]. The findings from this examination also demonstrated that taxi drivers declared a significantly higher degree of DA and aggressive driving in comparison with truck and bus drivers. These results support previous empirical findings [50,51] that taxi drivers had the tendency to take more risks and behave more aggressively than other professional drivers. This could be explained by the fact that taxi drivers operate without pre-defined lines and timetables, in contrast to other professional drivers. Therefore, due to daily job productivity, taxi drivers are prone to suffer from time pressure which, in turn, may provoke anger and aggressive behaviour during driving [53]. It is also possible that weak roadway design and traffic management can also lead to increased driving anger in the taxi drivers. For example, Mehri et al. [77] found that poor road infrastructure (narrow roads, shortage of parking spaces, and absence of taxi lanes), traffic jam, and a mixed traffic flow environment can evoke strong negative emotions in taxi drivers such as anger. Similar arguments were confirmed in other literature [78,79,80]. Identifying the differences between the categories of professional drivers may assist the decision-making process to improve the drivers’ safety performance. 

There are important constraints that may not be considered in the study and that should be highlighted. This research was conducted among professional drivers, and the results may not be generalizable to other groups of drivers. Therefore, further investigation including non-professional drivers is essential in the analysis and comprehension of the mechanisms of how drivers experience and express anger in an everyday traffic context. Secondly, Serbian, Montenegrin and Bosnian are almost identical to each other and belong to the family of Southern Slavic languages. Hence, the cross-language generalizability of the DAS should be examined by upcoming studies, taking into account various language families (e.g., Romance vs. Slavic). Thirdly, the present study was based on cross-sectional data collected from respondents at a single point in time. Accordingly, future research will be necessary to determine if the DA domains are invariant over time. Fourthly, data were collected at the beginning of the COVID-19 pandemic, which may have shaped drivers’ experiences and expressions of driving anger, and therefore, caution must be taken in trying to generalize these results. Hence, additional studies are needed to collect ongoing data to determine if these findings remain unchanged in the post−COVID period. Finally, a possible weakness associated to this study has to do with the quality pertaining to self-administered data, which can be influenced by lapses in memory, contextual factors and socially desirable responding. However, the participants were guaranteed that the answers they provided would be treated anonymously as well as confidentiality, which should minimize these biases. Nevertheless, this research offers initial evidence of the stability of the six-factor structure of the DAS instrument among professional drivers that may be beneficial during the investigation of the differences in DA across various cultural contexts. 

## 5. Conclusions

This study is one of the initial attempts when it comes to examining the factorial invariance of the original six-factor DAS model among professional drivers across culturally different countries. Therefore, from a theoretical point of view, this study replicates and extends the accumulated knowledge of previous investigations, suggesting that the original DAS remains a reliable and stable instrument for measuring DA in day-to-day driving conditions. The DAS facets also showed expected correlations with measures of aggressive driving behaviour and self-reported traffic violations and road accidents, confirming good convergent validity. Practically speaking, these findings enabled us to compare DA scores in professional drivers across the three countries and revealed that taxi drivers showed a more pronounced degree of DA and aggressive driving than truck and bus drivers. Moreover, because DA is considered an important emotional factor related to professional drivers’ safety performance [81], transportation companies may take advantage of these results by developing strategies for driver assessment and an effective road safety intervention programme. 

## Figures and Tables

**Table 1:** Demographic and driving characteristics of professional drivers by categories of driving licences in all the countries.

Demographic Variables		Driver Type			
		Taxi (*n* = 331)	Bus (*n* = 328)	Truck (*n* = 395)	Total (*n* = 1054)
Gender	Male	321 (97.0)	326 (99.4)	394 (99.7)	1041 (98.8)
	Female	10 (3.0)	2 (0.6)	1 (0.3)	13 (1.2)
Age	Mean (SD)	45.1 (11.2)	46.0 (10.1)	39.4 (10.1)	43.2 (10.9)
	18–34	60 (18.1)	43 (13.1)	137 (34.7)	240 (22.8)
	35–49	140 (42.3)	157 (47.9)	193 (48.9)	490 (46.5)
	50–64	125 (37.8)	116 (35.4)	60 (15.2)	301 (28.6)
	>65	6 (1.8)	12 (3.7)	5 (1.3)	23 (2.2)
Education	Primary school	3 (0.9)	15 (4.6)	11 (2.8)	29 (2.8)
	Secondary school	293 (88.5)	296 (90.2)	348 (88.1)	937 (88.9)
	Higher education	35 (10.6)	17 (5.2)	36 (9.1)	88 (8.3)
Experience	Mean (SD)	23.4 (10.7)	19.16 (10.7)	16.0 (10.5)	19.3 (11.0)
	0–5	17 (5.1)	38 (11.6)	73 (18.5)	128 (12.1)
	6–10	30 (9.1)	32 (9.8)	61 (15.4)	123 (11.7)
	11–15	40 (12.1)	65 (19.8)	81 (20.5)	186 (17.6)
	16>	244 (73.7)	193 (58.8)	180 (45.6)	617 (58.5)
Mileage	Mean (SD)	62,849.1 (29,358.3)	56,640.7 (39,608.9)	80,298.3 (40,334.7)	67,456.4 (38,353.8)
	0–10,000	5 (1.5)	16 (4.9)	7 (1.8)	28 (2.7)
	10,001–30,000	47 (15.7)	82 (25.0)	53 (13.4)	182 (17.3)
	30,001–60,000	114 (34.4)	114 (34.8)	68 (17.2)	396 (28.1)
	>60,000	165 (49.8)	116 (35.4)	267 (67.6)	548 (52.0)
Violations	Yes	150 (45.3)	160 (48.8)	160 (40.5)	470 (44.6)
	No	181 (54.7)	168 (51.2)	235 (59.5)	584 (55.4)
Accidents	Yes	222 (67.1)	231 (70.4)	289 (73.2)	742 (70.4)
	No	109 (32.9)	97 (29.6)	106 (26.8)	312 (29.6)

**Table 2:** Descriptive statistics, internal reliability analysis, Kruskal–Wallis tests and multivariate normality test statistics for study variables across countries.

		RS (*n* = 313)				MNE (*n* = 440)				B&H (*n* = 301)				Kruskal-Wallis Test
		Mdn	Mad	α	ω	Mdn	Mad	α	ω	Mdn	Mad	α	ω	χ^2^
DAS	Dis	2.75	0.93	0.85	0.85	2.63	1.11	0.90	0.90	2.88	1.11	0.87	0.87	6.24 *
	HG	2.33	0.99	0.84	0.84	2.00	0.99	0.81	0.81	2.00	1.48	0.78	0.79	2.62
	ID	2.25	1.11	0.77	0.75	2.75	1.11	0.76	0.76	2.50	1.11	0.73	0.73	12.53 **
	PP	1.50	0.74	0.74	0.75	1.25	0.37	0.83	0.82	1.25	0.37	0.75	0.75	5.11
	SD	2.00	0.74	0.83	0.82	2.00	0.99	0.81	0.82	2.00	0.74	0.83	0.83	0.52
	TO	2.57	1.05	0.85	0.85	2.29	0.86	0.79	0.79	2.29	0.84	0.77	0.76	31.66 ***
Mardia coefficient		1439.54 ***				1513.53 ***				1403.43 ***				
DAX-short	Ver	1.67	0.99	0.83	0.84	1.33	0.49	0.72	0.73	1.33	0.49	0.77	0.78	24.79 ***
	Phy	1.25	0.37	0.72	0.73	1.00	0.00	0.75	0.73	1.00	0.00	0.78	0.79	10.24 **
	Veh	1.67	0.98	0.70	0.70	1.33	0.49	0.57	0.57	1.67	0.98	0.57	0.56	12.31 **
	Adp	3.40	1.19	0.85	0.85	3.80	0.89	0.83	0.84	3.60	1.19	0.81	0.81	11.51 **
Mardia coefficient		348.34 ***				383.24 ***				368.23 ***				

Note. Mdn = Median; Mad = Median absolute deviation; α = Cronbach’s alpha coefficient; ω = Raykov’s omega coefficient; p(p+2) = 1155 for the DAS; p(p+2) = 255 for the DAX-short; χ^2^ = Chi-square; * *p* < 0.05, ** *p* < 0.01, *** *p* < 0.001.

**Table 3:** Fit indices for the six-factor model of the original DAS in RS, MNE and B&H.

	DWLS χ^2^	df	RMSEA	RMSEA CI	SRMR	CFI	TLI
RS	951.522 ***	480	0.056	[0.051, 0.061]	0.078	0.964	0.960
MNE	915.839 ***	480	0.045	[0.041, 0.050]	0.071	0.981	0.979
B&H	842.043 ***	480	0.050	[0.045, 0.056]	0.082	0.973	0.971

Note. χ^2^ = Chi-square; df = degree of freedom; RMSEA = Root Mean Square Error of Approximation; CI = Confidence Interval; SRMR = Standardized Root Mean Square Residual; CFI = Comparative Fit Index; TLI = Tucker-Lewis Index; *** *p* < 0.001.

**Table 4:** Standardized factor loadings for the original DAS in RS, MNE and B&H.

Item No.	Items	RS	MNE	B&H
	F1: Discourtesy			
5	Someone is driving very close to your rear bumper.	0.686	0.668	0.526
7	Someone cuts in right in front of you on the motorway.	0.601	0.713	0.709
8	Someone cuts in and takes the parking spot you have been waiting for.	0.641	0.714	0.643
12	Someone backs out right in front of you without looking.	0.605	0.671	0.704
14	Someone coming towards you does not dim their headlights at night.	0.577	0.751	0.655
15	At night someone is driving right behind you with bright lights on.	0.629	0.723	0.726
17	Someone speeds up when you try to pass them.	0.552	0.661	0.640
20	Someone pulls out right in front of you when there is no-one behind you.	0.715	0.692	0.657
32	A cyclist is riding in the middle of the lane and slowing traffic.	0.597	0.711	0.678
	F2: Hostile Gestures			
21	Someone makes an obscene gesture towards you about your driving.	0.782	0.836	0.812
23	Someone beeps at you about your driving.	0.799	0.782	0.729
26	Someone shouts at you about your driving.	0.814	0.679	0.671
	F3: Illegal Driving			
2	Someone is driving too fast for the road conditions.	0.530	0.625	0.610
6	Someone is weaving in and out of traffic.	0.817	0.755	0.728
13	Someone runs a red light or stop sign.	0.564	0.696	0.576
24	Someone is driving well above the speed limit.	0.720	0.578	0.659
	F4: Police Presence			
11	You see a police car watching traffic from a hidden position.	0.767	0.933	0.799
16	You pass a radar speed trap.	0.627	0.703	0.684
27	A police officer pulls you over.	0.567	0.574	0.530
33	A police car is driving in traffic close to you.	0.605	0.637	0.515
	F5: Slow Driving			
1	Someone in front of you does not move off straight away when the light turns to green.	0.690	0.467	0.540
3	A pedestrian walks slowly across the middle of the street, slowing you down.	0.678	0.508	0.502
4	Someone is driving too slowly in the outside lane, and holding up traffic.	0.699	0.757	0.746
9	Someone is driving more slowly than is reasonable for the traffic flow.	0.614	0.698	0.750
10	A slow vehicle on a winding road will not pull over and let people pass.	0.592	0.687	0.696
18	Someone is slow in parking and holds up traffic.	0.704	0.703	0.682
	F6: Traffic Obstruction			
19	You are stuck in a traffic jam.	0.714	0.683	0.631
22	You hit a deep pothole that was not marked.	0.536	0.584	0.594
25	You are driving behind a truck which has material flapping around in the back.	0.670	0.604	0.570
28	You are driving behind a vehicle that is smoking badly or giving off diesel fumes.	0.690	0.602	0.575
29	A truck kicks up sand or gravel on the car you are driving.	0.681	0.641	0.556
30	You are driving behind a large truck and cannot see around it.	0.778	0.599	0.511
31	You encounter road construction and detours.	0.585	0.370	0.422

**Table 5:** Measurement invariance of the original measurement model of the DAS across countries and types of professional drivers.

	DWLS χ^2^	df	CFI	RMSEA	ΔDWLS χ^2^	Δdf	*p*	ΔCFI	ΔRMSEA
Country									
Configural	2709.4	1440	0.975	0.050					
Metric	3463.4	1494	0.961	0.061	753.96	54	<0.001	0.014	0.011
Scalar	3692.1	1548	0.957	0.063	228.72	54	<0.001	0.004	0.002
Residual	3888.5	1614	0.954	0.063	196.45	66	<0.001	0.003	0.001
Types of professional drivers									
Configural	3007.5	1440	0.964	0.056					
Metric	3498.9	1494	0.954	0.062	491.47	54	<0.001	0.010	0.006
Scalar	3576.9	1548	0.954	0.061	77.93	54	<0.05	0.001	0.001
Residual	3907.1	1614	0.948	0.064	330.20	66	<0.001	0.006	0.003

**Table 6:** Spearman’s correlation coefficients among study variables (N = 1054).

		Dis	HG	ID	PP	SD	TO	Ver	Phy	Veh	Adp	Vio	Acc
DAS	Dis	1	-	-	-	-	-	-	-	-	-	-	-
	HG	0.55 ***	1	-	-	-	-	-	-	-	-	-	-
	ID	0.56 ***	0.34 ***	1	-	-	-	-	-	-	-	-	-
	PP	0.35 ***	0.37 ***	0.17 ***	1	-	-	-	-	-	-	-	-
	SD	0.70 ***	0.49 ***	0.35 ***	0.53 ***	1	-	-	-	-	-	-	-
	TO	0.69 ***	0.58 ***	0.43 ***	0.49 ***	0.66 ***	1	-	-	-	-	-	-
DAX-short	Ver	0.29 ***	0.26 ***	0.08 *	0.35 ***	0.40 ***	0.34 ***	1	-	-	-	-	-
	Phy	0.21 ***	0.26 ***	0.07 *	0.40 ***	0.37 ***	0.32 ***	0.60 ***	1	-	-	-	-
	Veh	0.29 ***	0.25 ***	0.05	0.36 ***	0.40 ***	0.31 ***	0.56 ***	0.55 ***	1	-	-	-
	Adp	0.01	−0.08 *	0.08 **	−0.24 ***	−0.10 **	−0.05	−0.25 ***	−0.28 ***	−0.19 ***	1	-	-
	Vio	0.10 **	0.06	0.01	0.12 ***	0.12 ***	0.09 **	0.15 ***	0.11 ***	0.13 ***	−0.02	1	-
	Acc	0.01	0.06 *	−0.04	0.05	0.06	0.04	0.09 **	0.06 *	0.04	−0.02	0.20 ***	1

Note.; Vio = self-reported traffic violations; Acc = self-reported road accidents; * *p* < 0.05, ** *p* < 0.01, *** *p* < 0.001.

**Table 7:** Descriptive statistics and Kruskal–Wallis tests for study variables across professional driver types.

		Taxi (*n* = 331)		Bus (328)		Truck (395)		Kruskal-Wallis Test
		Mdn	Mad	Mdn	Mad	Mdn	Mad	χ^2^
DAS	Dis	3.13	0.93	2.38	0.93	2.75	0.93	65.88 ***
	HG	2.33	1.48	1.67	0.99	2.33	0.99	24.85 ***
	ID	2.50	1.11	2.25	1.11	2.75	1.11	27.41 ***
	PP	1.50	0.74	1.25	0.37	1.50	0.74	67.59 ***
	SD	2.33	0.98	1.67	0.74	2.00	0.74	97.45 ***
	TO	2.71	0.86	1.86	0.64	2.29	0.85	86.40 ***
DAX-short	Ver	1.67	0.99	1.33	0.49	1.33	0.49	34.60 ***
	Phy	1.25	0.37	1.00	0.00	1.00	0.00	23.00 ***
	Veh	1.67	0.98	1.33	0.49	1.33	0.49	27.47 ***
	Adp	3.40	1.19	3.80	1.04	3.60	1.19	6.76 *

Note. Mdn = Median; Mad = Median absolute deviation; χ^2^ = Chi-square; * *p* < 0.05, *** *p* < 0.001.

## Data Availability

Not applicable.

## Supplementary Materials

The following supporting information can be downloaded at: https://www.mdpi.com/article/10.3390/ijerph19074168/s1, Table S1: Demographic and driving characteristics of professional drivers by categories of driving licenses in Serbia; Table S2: Demographic and driving characteristics of professional drivers by categories of driving licenses in Montenegro; Table S3: Demographic and driving characteristics of professional drivers by categories of driving licenses in Bosnia and Herzegovina; Table S4: Fit indices of the short version of DAX in Serbia, Montenegro and Bosnia and Herzegovina; Table S5: Standardized item-factor loadings for the short version of DAX in Serbia, Montenegro and Bosnia and Herzegovina; Table S6: Measurement invariance of the four-factor model of the short version of DAX across countries and types of professional drivers.; Table S7: Univariate descriptive statistics of the DAS and the DAX-short scales by types of professional drivers in Serbia; Table S8: Univariate descriptive statistics of the DAS and the DAX-short scales by types of professional drivers in Montenegro; Table S9: Univariate descriptive statistics of the DAS and the DAX-short scales by types of professional drivers in Bosnia and Herzegovina.
