# Supplementary material for: Differences in Driving Anger among Professional Drivers: A Cross-Cultural Study
Source: Int J Environ Res Public Health. 2022 Mar 31;19(7):4168. doi: 10.3390/ijerph19074168 (PMC8999064; doi:10.3390/ijerph19074168)
Supplement: Supplementary file 1 [file ijerph-19-04168-s001.zip › ijerph-1625867-supplementary.pdf]

Supplementary Material

Table S1. Demographic and driving characteristics of professional drivers by categories of driving licenses in Serbia

Demographic variables		Driver type			
		**Taxi (n=102)**	**Bus (n=102)**	**Truck (n=109)**	**Total (n=313)**
Gender	Male	102 (100.0)	101 (99.0)	109 (100.0)	312 (99.7)
	Female	0 (0.0)	1 (1.0)	0 (0.0)	1 (0.3)
Age	Mean (SD)	49.9 (8.2)	47.3 (8.6)	38.0 (7.7)	44.9 (9.7)
	18 - 34	5 (4.9)	6 (5.9)	38 (34.9)	49 (15.7)
	35 - 49	41 (40.2)	53 (52.0)	62 (56.9)	156 (49.8)
	50 - 64	54 (52.9)	40 (39.2)	9 (8.3)	103 (32.9)
	65+	2 (2.0)	3 (2.9)	0 (0.0)	5 (1.6)
Educational attainment	Primary school	2 (2.0)	3 (2.9)	2 (1.8)	7 (2.2)
	Secondary school	89 (87.3)	93 (91.2)	92 (84.4)	274 (87.5)
	Higher education	11 (10.8)	6 (5.9)	15 (5.9)	32 (10.2)
Years of professional driving experience	Mean (SD)	26.8 (8.2)	20.5 (9.5)	15.2 (8.7)	20.7 (10.0)
	0 - 5	0 (0.0)	8 (7.8)	17 (15.6)	25 (8.0)
	6 - 10	1 (1.0)	6 (5.9)	21 (19.3)	28 (8.9)
	11 - 15	13 (12.7)	21 (20.6)	22 (20.2)	56 (17.9)
	16 and above	88 (86.3)	67 (65.7)	49 (45.0)	204 (65.2)
Annual mileage	Mean (SD)	73676.5 (24981.2)	46299.0 (26378.7)	93572.5 (37509.2)	71683.4 (35967.2)
	0 - 10.000	0 (0.0)	3 (2.9)	0 (0)	3 (1.0)
	10.001 - 30.000	3 (2.9)	16 (15.7)	4 (3.7)	23 (7.3)
	30.001 - 60.000	37 (36.3)	65 (63.7)	20 (18.3)	122 (39.0)
	60.000 and above	62 (60.8)	18 (17.6)	85 (78.0)	165 (52.7)
Involvement in a driving violation in the last 3 years (self-reported)	Yes	55 (53.9)	66 (64.7)	62 (56.9)	183 (58.5)
	No	47 (46.1)	36 (35.3)	47 (43.1)	130 (41.5)
Involvement in a road accident in the last 3 years (self-reported)	Yes	61 (59.8)	57 (55.9)	75 (68.8)	193 (61.7)
	No	41 (40.2)	45 (44.1)	34 (31.2)	120 (38.3)

Table S2. Demographic and driving characteristics of professional drivers by categories of driving licenses in Montenegro

Demographic variables		Driver type			
		Taxi (n=128)	Bus (n=126)	Truck (n=186)	Total (n=440)
Gender	Male	118 (92.2)	126 (100.0)	185 (99.5)	429 (97.5)
	Female	10 (7.8)	0 (0.0)	1 (0.5)	11 (2.5)
Age	Mean (SD)	44.1 (11.2)	46.3 (11.3)	40.0 (10.7)	43.0 (11.4)
	18 - 34	26 (20.3)	20 (15.9)	65 (34.9)	111 (25.2)
	35 - 49	58 (45.3)	55 (43.7)	85 (45.7)	198 (45.0)
	50 - 64	41 (32.0)	43 (34.1)	33 (17.7)	117 (26.6)
	65+	3 (2.3)	8 (6.3)	3 (1.6)	14 (3.2)
Educational attainment	Primary school	1 (0.8)	11 (8.7)	9 (4.8)	21 (4.8)
	Secondary school	109 (85.2)	107 (84.9)	159 (85.5)	375 (85.2)
	Higher education	18 (14.1)	8 (6.3)	18 (9.7)	44 (10.0)
Years of professional driving experience	Mean (SD)	23.2 (10.9)	20.3 (11.2)	17.1 (11.2)	19.8 (11.4)
	0 - 5	8 (6.3)	11 (8.7)	32 (17.2)	51 (11.6)
	6 - 10	11 (8.6)	17 (13.5)	28 (15.1)	56 (12.7)
	11 - 15	14 (10.9)	23 (18.3)	34 (18.3)	71 (16.1)
	16 and above	95 (74.2)	75 (59.5)	92 (49.5)	262 (59.5)
Annual mileage	Mean (SD)	48997.2 (30966.7)	52959.5 (43552.4)	63941.9 (42850.1)	56449.4 (40432.0)
	0 - 10.000	5 (3.9)	10 (7.9)	7 (3.8)	22 (5.0)
	10.001 - 30.000	37 (28.9)	49 (38.9)	48 (25.8)	134 (30.5)
	30.001 - 60.000	52 (40.6)	22 (7.5)	45 (24.2)	119 (27.0)
	60.000 and above	34 (26.6)	45 (35.7)	86 (46.2)	165 (37.5)
Involvement in a driving violation in the last 3 years (self-reported)	Yes	52 (40.6)	39 (31.0)	61 (32.8)	152 (34.5)
	No	76 (59.4)	87 (69.0)	125 (67.2)	288 (65.5)
Involvement in a road accident in the last 3 years (self-reported)	Yes	89 (69.5)	96 (76.2)	145 (78.0)	330 (75.0)
	No	39 (30.5)	30 (23.8)	41 (22.0)	110 (25.0)

Table S3. Demographic and driving characteristics of professional drivers by categories of driving licenses in Bosnia and Herzegovina

Demographic variables		Driver type			
		Taxi (n=101)	Bus (n=100)	Truck (n=100)	Total (n=301)
Gender	Male	101 (100.0)	99 (99.0)	100 (100.0)	299 (99.7)
	Female	0 (0.0)	1 (1.0)	0 (0.0)	1 (0.3)
Age	Mean (SD)	41.7 (12.3)	44.3 (9.8)	39.6 (11.0)	41.8 (11.2)
	18 - 34	29 (28.7)	17 (17.0)	34 (34.0)	80 (26.6)
	35 - 49	41 (40.6)	49 (49.0)	46 (46.0)	136 (45.2)
	50 - 64	30 (29.7)	33 (33.0)	18 (18.0)	81 (36.9)
	65+	1 (1.0)	1 (1.0)	2 (2.0)	4 (1.3)
Educational attainment	Primary school	0 (0.0)	1 (1.0)	0 (0.0)	1 (0.3)
	Secondary school	95 (94.1)	96 (96.0)	97 (97.0)	288 (95.7)
	Higher education	6 (5.9)	3 (3.0)	3 (3.0)	12 (4.0)
Years of professional driving experience	Mean (SD)	20.2 (11.8)	16.3 (10.9)	15.0 (10.8)	17.2 (11.3)
	0 - 5	9 (8.9)	19 (19.0)	24 (24.0)	52 (17.3)
	6 - 10	18 (17.8)	9 (9.0)	12 (12.0)	39 (13.0)
	11 - 15	13 (12.9)	21 (21.0)	25 (25.0)	59 (19.6)
	16 and above	61 (60.4)	51 (51.0)	39 (39.0)	151 (50.2)
Annual mileage	Mean (SD)	69469.4 (24221.9)	71827.5 (41433.6)	96252.5 (23557.5)	79150.9 (33047.7)
	0 - 10.000	0 (0.0)	3 (3.0)	0 (0.0)	3 (1.0)
	10.001 - 30.000	7 (6.9)	17 (17.0)	1 (1.0)	25 (8.3)
	30.001 - 60.000	25 (24.8)	27 (27.0)	3 (3.0)	55 (18.3)
	60.000 and above	69 (68.3)	53 (53.0)	96 (96.0)	218 (72.4)
Involvement in a driving violation in the last 3 years (self-reported)	Yes	43 (42.6)	55 (55.0)	37 (37.0)	135 (44.9)
	No	58 (57.4)	45 (45.0)	63 (63.0)	166 (55.1)
Involvement in a road accident in the last 3 years (self-reported)	Yes	72 (71.3)	78 (78.0)	69 (69.0)	219 (72.8)
	No	29 (28.7)	22 (22.0)	31 (31.0)	82 (27.2)

Table S4. Fit indices of the short version of DAX in Serbia, Montenegro and Bosnia and Herzegovina

	DWLS χ^2	*df*	*RMSEA*	*RMSEA CI*	*SRMR*	*CFI*	*TLI*
Serbia (*n*=313)	99.308	84	0.024	[0.000, 0.041]	0.059	0.995	0.994
Montenegro (*n*=440)	128.888***	84	0.035	[0.022, 0.046]	0.054	0.983	0.979
Bosnia and Herzegovina (*n*=301)	98.803	84	0.023	[0.000, 0.041]	0.055	0.992	0.990

Note. χ^2 = Chi-square; *df* = degree of freedom; *RMSEA* = Root Mean Square Error of Approximation; *CI* = Confidence Interval; *SRMR* = Standardized Root Mean Square Residual; *CFI* = Comparative Fit Index; *TLI* = Tucker-Lewis Index; $^*p < 0.05$, $^{**}p < 0.01$, $^{***}p < 0.001$

Table S5. Standardized item-factor loadings for the short version of DAX in Serbia, Montenegro and Bosnia and Herzegovina

**Item no.**	**Items**	**Serbia**	**Montenegro**	**Bosnia and Herzegovina**
	**F1: Verbal Aggressive Expression**			
6	I make negative comments about the other driver	0.640	0.668	0.680
28	I swear at the other driver aloud.	0.834	0.722	0.772
38	I yell at the other driver.	0.890	0.663	0.732
	**F2: Physical Aggressive Expression**			
8	I try to get out of the car and tell the other driver off.	0.576	0.664	0.683
10	I roll down the window to help communicate my anger.	0.644	0.673	0.811
21	I try to scare the other driver.	0.715	0.684	0.730
41	I try to get out of the car and have a physical fight with the other driver.	0.607	0.562	0.559
	**F3: Using the Vehicle for Aggressive Expression**			
2	I drive right up on the other driver's bumper.	0.630	0.641	0.611
22	I do to other drivers what they did to me.	0.699	0.550	0.561
27	I drive a lot faster than I was.	0.654	0.504	0.499
	**F4: Adaptive/Constructive Expression**			
26	I try to think of positive solutions to deal with the situation.	0.675	0.723	0.699
29	I tell myself it is not worth getting all mad about.	0.702	0.721	0.644
36	I tell myself it's not worth getting involved in.	0.832	0.767	0.778
45	I just try and accept that there are frustrating situations while driving.	0.661	0.540	0.530
48	I tell myself to ignore it.	0.760	0.782	0.712

Note. *n* =313 Serbian; *n* = 440 Montenegrin; n = 301 Bosnian

Table S6. Measurement invariance of the four-factor model of the short version of DAX across countries and types of professional drivers.

	DWLS χ²	*df*	*CFI*	*RMSEA*	*ΔDWLS χ²*	*Δdf*	*p*	*ΔCFI*	*ΔRMSEA*
**Country**									
Configural	325.00	252	0.990	0.029					
Metric	392.35	274	0.983	0.035	67.35	22	< 0.001	0.006	0.006
Scalar	456.41	296	0.978	0.039	64.06	22	< 0.001	0.006	0.004
Residual	522.53	326	0.972	0.041	66.12	30	< 0.001	0.005	0.002
**Types of professional drivers.**									
Configural	314.15	252	0.991	0.027					
Metric	463.23	274	0.973	0.044	149.08	22	< 0.001	0.018	0.018
Scalar	519.82	296	0.968	0.046	56.59	22	< 0.001	0.005	0.002
Residual	609.44	326	0.959	0.050	89.62	30	< 0.001	0.009	0.003

Table S7. Univariate descriptive statistics of the DAS and the DAX-short scales by types of professional drivers in Serbia

		**Taxi**				**Bus**				**Truck**			
		Mean	*SD*	*S*	*K*	*Mean*	*SD*	*S*	*K*	*Mean*	*SD*	*S*	*K*
DAS	Dis	3.21	0.66	-0.16	-0.31	2.33	0.83	0.79	-0.04	2.79	0.73	0.40	-0.68
	HG	2.34	1.05	0.36	-0.81	1.87	0.90	1.04	0.33	2.76	0.94	0.47	-0.15
	ID	2.43	0.98	0.33	-0.75	2.30	1.04	0.83	-0.01	2.62	0.73	0.45	0.19
	PP	1.91	0.70	0.86	0.00	1.19	0.42	3.86	20.52	1.86	0.65	0.46	-0.72
	SD	2.75	0.72	0.08	-0.16	1.61	0.59	1.49	2.53	2.02	0.61	1.30	1.77
	TO	3.14	0.73	-0.48	-0.59	1.86	0.62	1.31	2.05	2.80	0.75	0.19	-0.47
DAX-short	Ver	2.32	1.06	0.35	-1.00	1.55	0.67	1.27	1.23	1.91	0.75	0.71	0.00
	Phy	1.63	0.68	1.21	1.20	1.18	0.46	3.64	14.17	1.46	0.58	1.42	1.62
	Veh	2.10	0.85	0.28	-0.83	1.35	0.46	1.57	2.64	1.65	0.62	1.13	1.11
	Adp	3.35	0.97	0.15	-0.77	3.69	0.93	-0.42	-0.22	3.18	1.16	-0.35	-1.09

Note. *SD* = Standard Deviation; *S* = Skewness, *K* = Kurtosis

Table S8. Univariate descriptive statistics of the DAS and the DAX-short scales by types of professional drivers in Montenegro

		**Taxi**				**Bus**				**Truck**			
		Mean	*SD*	*S*	*K*	*Mean*	*SD*	*S*	*K*	*Mean*	*SD*	*S*	*K*
DAS	Dis	2.95	0.96	-0.10	-0.74	2.42	0.76	0.22	-0.81	2.79	0.98	0.42	-0.65
	HG	2.65	1.18	0.26	-0.99	2.15	0.94	0.51	-0.94	2.10	1.06	0.94	0.04
	ID	2.74	1.08	0.10	-0.88	2.44	0.95	0.49	-0.04	2.87	1.01	-0.25	-0.88
	PP	1.87	0.91	1.29	1.33	1.51	0.60	1.48	1.93	1.54	0.82	1.82	2.99
	SD	2.34	0.88	0.36	-0.58	1.94	0.68	0.97	0.49	2.02	0.79	0.87	0.68
	TO	2.47	0.84	0.39	0.13	2.21	0.71	0.31	-1.05	2.21	0.75	0.70	0.45
DAX-short	Ver	1.78	0.88	0.94	-0.23	1.55	0.64	1.07	0.41	1.51	0.66	1.37	1.33
	Phy	1.46	0.63	1.34	0.88	1.38	0.60	1.94	3.16	1.35	0.60	2.26	4.90
	Veh	1.66	0.61	0.80	-0.06	1.59	0.66	1.14	0.42	1.47	0.66	1.69	2.82
	Adp	3.46	1.13	-0.75	-0.37	3.63	0.94	-0.63	-0.13	3.58	1.06	-0.92	0.07

Note. *SD* = Standard Deviation; *S* = Skewness, *K* = Kurtosis

Table S9. Univariate descriptive statistics of the DAS and the DAX-short scales by types of professional drivers in Bosnia and Herzegovina

		Taxi				Bus				Truck			
		**Mean**	**SD**	**S**	**K**	**Mean**	**SD**	**S**	**K**	**Mean**	**SD**	**S**	**K**
DAS	Dis	2.97	0.98	-0.13	-1.06	2.73	0.93	0.22	-0.73	2.97	0.83	0.10	-0.41
	HG	2.32	1.22	0.61	-0.75	2.02	1.00	1.10	0.92	2.35	1.04	0.48	-0.55
	ID	2.72	1.03	0.58	-0.39	2.37	0.96	0.32	-0.84	2.65	1.02	0.33	-0.62
	PP	1.64	0.81	1.48	1.50	1.36	0.48	1.69	2.78	1.73	0.74	1.39	1.68
	SD	2.18	0.83	0.85	0.56	1.85	0.71	1.33	2.50	2.25	0.82	0.67	0.01
	TO	2.42	0.91	0.48	-0.26	2.06	0.61	0.08	-0.98	2.27	0.66	0.33	0.01
DAX-short	Ver	1.80	0.88	1.39	1.90	1.42	0.56	1.58	2.54	1.91	0.86	0.61	-0.66
	Phy	1.32	0.65	2.41	5.20	1.20	0.37	2.00	3.12	1.47	0.70	1.62	2.18
	Veh	1.67	0.80	1.64	2.67	1.54	0.60	1.05	0.30	1.98	0.72	0.43	0.00
	Adp	3.32	1.07	-0.53	-0.46	3.43	1.05	-0.59	-0.52	3.35	0.88	-0.13	-0.16

Note. *SD* = Standard Deviation; *S* = Skewness, *K* = Kurtosis
